# *OsVTC1-1* Gene Silencing Promotes a Defense Response in Rice and Enhances Resistance to *Magnaporthe oryzae*

**DOI:** 10.3390/plants11172189

**Published:** 2022-08-24

**Authors:** Kanyanat Lamanchai, Nicholas Smirnoff, Deborah L. Salmon, Athipat Ngernmuen, Sittiruk Roytrakul, Kantinan Leetanasaksakul, Suthathip Kittisenachai, Chatchawan Jantasuriyarat

**Affiliations:** 1 Department of Genetics, Faculty of Science, Kasetsart University, Chatuchak, Bangkok 10900, Thailand; 2 Biosciences, College of Life and Environmental Sciences, University of Exeter, Exeter EX4 4QD, UK; 3 Department of Zoology, Faculty of Science, Kasetsart University, Chatuchak, Bangkok 10900, Thailand; 4 Functional Proteomics Technology, National Center for Genetic Engineering and Biotechnology, National Science and Technology Development Agency, 113 Paholyothin Road, Klong 1, Klong Luang, Pathumthani 12120, Thailand; 5 Center for Advanced Studies in Tropical Natural Resources, National Research University-Kasetsart (CASTNAR, NRU-KU), Kasetsart University, Chatuchak, Bangkok 10900, Thailand

**Keywords:** GDP-D-mannose pyrophosphorylase, ascorbic acid, *Magnaporthe oryzae*, rice blast

## Abstract

Rice blast disease is a serious disease in rice caused by *Magnaporthe oryzae* (*M. oryzae*). Ascorbic acid (AsA), or vitamin C, is a strong antioxidant that prevents oxidative damage to cellular components and plays an essential role in plant defense response. GDP-D-mannose pyrophosphorylase (GMP or VTC1) is an enzyme that generates GDP-D-mannose for AsA, cell wall, and glycoprotein synthesis. The *OsVTC1* gene has three homologs in the rice genome: *OsVTC1-1*, *OsVTC1-3*, and *OsVTC1-8*. Using *OsVTC1-1* RNAi lines, this study investigated the role of the *OsVTC1-1* gene during rice blast fungus inoculation. The *OsVTC1-1* RNAi inoculated with rice blast fungus induced changes to cell wall monosaccharides, photosynthetic efficiency, reactive oxygen species (ROS) accumulation, and malondialdehyde (MDA) content. Additionally, the *OsVTC1-1* RNAi lines were shown to be more resistant to rice blast fungus than the wild type. Genes and proteins related to defense response, plant hormone synthesis, and signaling pathways, especially salicylic acid and jasmonic acid, were up-regulated in the *OsVTC1-1* RNAi lines after rice blast inoculation. These results suggest that the *OsVTC1-1* gene regulates rice blast resistance through several defense mechanisms, including hormone synthesis and signaling pathways.

## 1. Introduction

Rice blast is one of the most devastating diseases affecting rice production worldwide. It is caused by a filamentous ascomycete fungus, *Magnaporthe oryzae* (*M. oryzae*) [1]. Rice blast fungus causes lesions on all aboveground plant physiology, including the leaves, leaf collars, necks, panicles, and seeds. The symptoms on leaves appear as diamond-shaped lesions, initially, but then the lesions extend and kill the entire leaf [2]. There are several strategies that plants employ to detect and combat invading pathogens, such as impenetrable barriers, phytoalexins, callose deposition, and production of pathogenesis-related (PR) proteins [3,4,5]. An efficient mechanism to prevent pathogen invasion is hypersensitivity response (HR), which results in rapid cell death at the pathogen attack site to restrict pathogen invasion [6,7,8]. During HR, reactive oxygen species (ROS) are rapidly generated and cause oxidative damage through interactions with nucleic acids, proteins, and lipids [9,10,11].

A potent antioxidant, ascorbic acid (AsA), also known as vitamin C, plays an essential role in many biological processes, such as participating as an enzyme cofactor, photosynthesis, cell wall growth, cell expansion, and response to biotic and abiotic stresses [12,13,14]. Plants synthesize AsA primarily via the L-galactose pathway or the Smirnoff-Wheeler pathway [15,16]. Enzymes in the L-galactose pathway consist of phosphoglucose isomerase (PGI), phosphomannose isomerase (PMI), phosphomannose mutase (PMM), GDP-mannose pyrophosphorylase (GMP or VTC1), GDP-mannose-3′,5′-epimerase (GME), GDP-L-galactose phosphorylase (GGP or VTC2), L-galactose-1-phosphate phosphatase (GPP or VTC4), L-galactose dehydrogenase (GDH), and L-galactono-1,4-lactone dehydrogenase (GLDH). However, VTC1 catalyzes the formation of GDP-D-mannose, which serves as a precursor for AsA synthesis and is critical for cell wall polysaccharide and glycoprotein synthesis [17]. There are three *OsVTC1* homologous genes in the rice genome: *OsVTC1-1*, *OsVTC1-3*, and *OsVTC1-8*. AsA content is increased in *OsVTC1-1* and *OsVTC1-3* overexpression lines and not *OsVTC1-8* overexpression lines, indicating that *OsVTC1-1* and *OsVTC1-3* are involved in plant AsA synthesis. *OsVTC1-1* and *OsVTC1-3* are involved in the production of AsA in rice leaves and roots, respectively [18]. Several studies have investigated the function of *VTC1* gene in plants. Down-regulation of *OsVTC1-1* genes led to increased superoxide (O_2_^−^) anion and malondialdehyde (MDA) levels as well as decreased chlorophyll levels as a result of decreased salt tolerance [19]. A previous study also showed that the overexpression of tomato *GMPase* (*VTC1*) increased the AsA content in tobacco and led to increased thermal tolerance [20]. Barth et al. [21] reported that bacterial and fungal hyphal development was suppressed in *vtc1 Arabidopsis* mutants with lower levels of AsA. Similarly, the presence of dead cells and an increased resistance to *Pseudomonas syringae* infection in *Arabidopsis* were observed in *vtc1* mutants [22]. These studies indicate that *VTC1* is vital in plant responses to abiotic and biotic stresses.

Many biological processes are altered after pathogen infection [23]. Upon infection with a biotrophic fungal pathogen, plants lose their ability to photosynthesize and mobilize nutrients [24]. Cell walls, as a physical barrier, are the first plant defense mechanism against pathogens. Upon infection, plants accumulate reinforcing polymers, such as callose and phenolic compounds, for cell wall strengthening [25,26]. High sugar content in plant tissues is associated with plant resistance, serving as substrates for cell wall polymers, signaling molecules, and energy for defense responses [27,28]. Furthermore, ROS accumulation in the plant’s subcellular compartments, which is toxic to the cells, acts as signaling molecules during defense responses [29]. A previous study reported that ROS accumulation and cell death were observed in resistant accessions after infection with *Alternaria brassicae* in *Arabidopsis* [30]. ROS also promote lipid peroxidation, detectable by measuring malondialdehyde (MDA) content [31]. In incompatible rice and *M. oryzae* interactions, ROS accumulation and increased MDA content were measured, which ultimately resulted in HR cell death [32]. Additionally, plant hormones are pivotal in regulating plant defense against various pathogens [33]. Among several plant hormones, salicylic acid (SA) and jasmonic acid (JA) are key in defense against insect herbivores and microbial pathogens [34].

Although, the above studies revealed that pathogen infections induce several defense mechanisms in plants, it remains unclear how AsA synthesis is affected during rice defense responses. *OsVTC1-1* in the AsA synthesis pathway might affect defenses against rice blast infections through several processes. Therefore, the aim of the present study was to investigate the role of *OsVTC1-1* during rice blast infection. We compared cell wall monosaccharide composition, photosynthetic efficiency, AsA content, superoxide anion (O_2_^−^) levels, malondialdehyde (MDA) content, transcriptome, and proteome between wild type (WT) and *OsVTC1-1* RNAi lines after rice blast fungus infection. *OsVTC1-1* RNAi rice seeds, which knock-down the *OsVTC1-1* gene, were obtained from Qin et al. [18]. Due to the antioxidant properties of AsA, the reduction in *OsVTC1-1* gene expression should improve the defense of plants against rice blast infections. Reducing AsA levels increases ROS and MDA accumulation in HR cell death and protects photosynthesis machinery. In addition, transcriptome and proteome profiles should alter in the *OsVTC1-1* RNAi line as the *OsVTC1-1* RNAi lines enhance resistance to rice blast fungus infections. Several genes and proteins were differentially expressed, especially those involved in defense mechanisms, hormone synthesis, and signaling pathways. These results demonstrate the important role of *OsVTC1-1* in rice defense responses during rice blast fungus infections.

## 2. Results

### 2.1. Pathogenicity Assay

**Figure 1:**
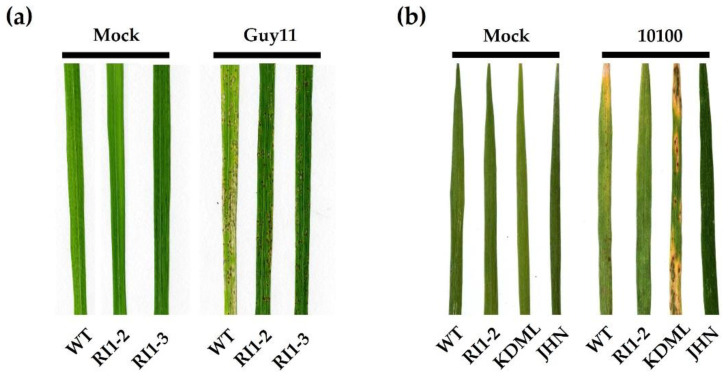
Disease symptoms on wild-type (WT) and *OsVTC1-1* RNAi lines (RI1-2 and RI1-3) rice leaves inoculated with rice blast fungus isolates. (**a**) Guy11; (**b**) 10100, Khao Dawk Mali 105 (KDML105) and Jao Hom Nin (JHN) were used as susceptible and resistant controls, respectively. Diseased rice leaves were photographed 7 days post inoculation.

Rice blast disease symptoms were observed 7 days after inoculation. Lesions were observed in both WT and *OsVTC1-1* RNAi lines caused by the rice blast Guy11 isolate. RI1-2 and RI1-3 lines, however, had smaller and fewer lesions than WT (Figure 1a). Similar results were observed in leaves infected with rice blast 10100 isolate from the RI1-2 line. Khao Dawk Mali 105 (KDML105) and Jao Hom Nin (JHN) were used as susceptible and resistant controls, respectively (Figure 1b). A mock inoculation showed no symptoms on leaves (Figure 1). The results show that *OsVTC1-1* RNAi lines have enhanced disease resistance against rice blast fungus.

### 2.2. Cell Wall Sugar Composition Analysis

**Figure 2:**
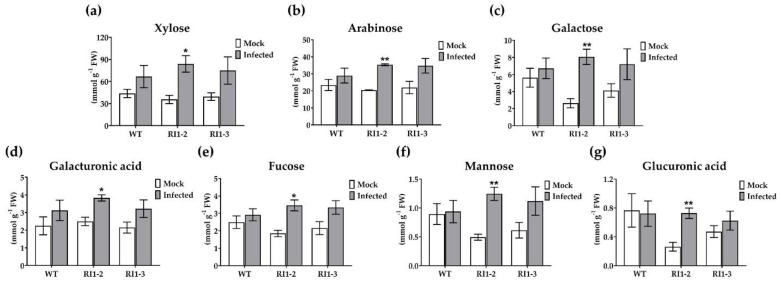
Cell wall monosaccharide composition (mmol g^−1^ fresh weight) of 3-week-old rice leaves from wild-type (WT) and *OsVTC1-1* RNAi lines at 4 days after inoculation with Guy11 isolate. (**a**) Xylose; (**b**) arabinose; (**c**) galactose; (**d**) galacturonic acid; (**e**) fucose; (**f**) mannose; (**g**) glucuronic acid. Data are presented as the mean ± standard error of three replicates. Asterisk indicates statistically significant differences between mock and infected plants for each rice line analyzed using Student’s *t*-test (* *p* < 0.05 and ** *p* < 0.01).

To assess whether the *OsVTC1-1* gene is involved in a cell-wall-associated defense mechanism against rice blast fungus, the composition of cell walls at 4 days after inoculation with rice blast fungus in WT and *OsVTC1-1* RNAi lines was investigated. Xylose, arabinose, galactose, galacturonic acid, fucose, mannose, and glucuronic acid were measured as monosaccharides in the cell wall. The highest proportion of xylose was found in hydrolyzed cell wall samples, followed by arabinose and galactose (Figure 2a–c). Comparison between WT rice and the *OsVTC1-1* RNAi line in a mock inoculation was previously reported [35], in which the *OsVTC1-1* RNAi line showed lower levels of galactose, mannose, and glucuronic acid than WT rice (Figure 2c,f,g). After rice blast inoculation, cell wall monosaccharide compositions were increased in both WT rice and the *OsVTC1-1* RNAi line (Figure 2a–g). Moreover, all cell wall monosaccharide compositions were significantly higher in the *OsVTC1-1* RNAi line than in the WT after rice blast fungus infection (Figure 2a–g). These results suggest that *OsVTC1-1* is involved in monosaccharide deposition in the cell wall during rice blast infection.

### 2.3. Photosynthetic Efficiency Analysis

**Figure 3:**
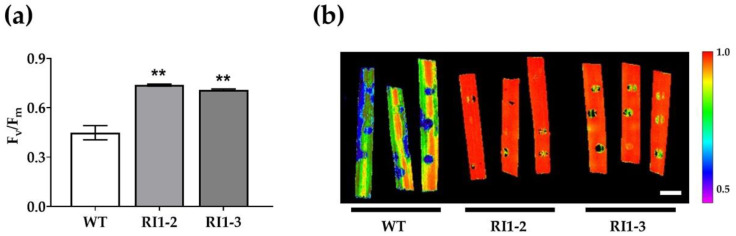
Leaf photosynthetic efficiency in wild-type (WT) and *OsVTC1-1* RNAi lines (RI1-2 and RI1-3) at 5 days post inoculation with Guy11 isolate. (**a**) Maximal efficiency of PSII (F_v_/F_m_); (**b**) chlorophyll fluorescence image showing photosynthetic activity across the leaf surface (scale bar = 1 cm). Data are presented as the mean ± standard error of three replicates. Asterisk indicates statistically significant differences from WT analyzed using Student’s *t*-test (** *p* < 0.01).

Maximum quantum efficiency of photosystem II (PSII) was measured by the F_v_/F_m_ parameter and used to assess chlorophyll fluorescence to compare photosynthetic efficiency between WT and *OsVTC1-1* RNAi lines at 5 days post inoculation. The *OsVTC1-1* RNAi lines showed higher maximum efficiency of PSII (F_v_/F_m_) in infected leaves compared to the WT (Figure 3a). According to fluorescence imaging of chlorophyll fluorescence, the photosynthetic system of WT plants was affected by *M. oryzae* infection, but *OsVTC1-1* RNAi plants were not affected (Figure 3b). These results suggest that the reduction in *OsVTC1-1* gene expression protected photosynthetic efficiency in *OsVTC1-1* RNAi lines during *M. oryzae* infection. 

### 2.4. Measurement of Ascorbic Acid Content

**Figure 4:**
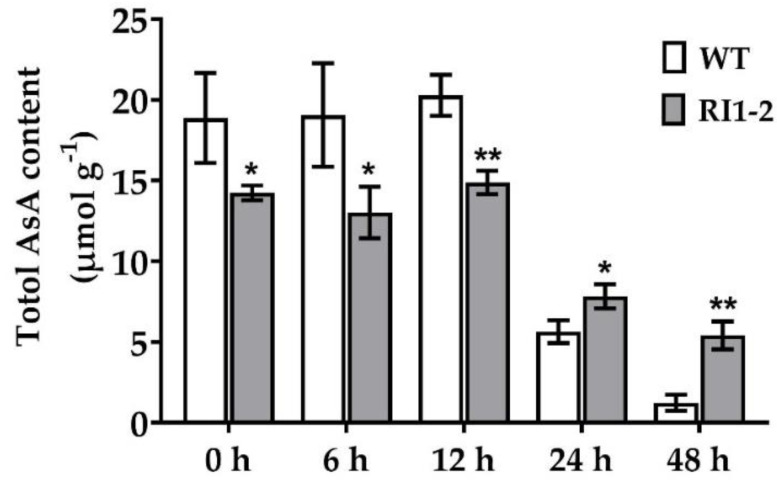
Ascorbic acid content in wild-type (WT) and *OsVTC1-1* RI1-2 line after rice fungus inoculation with 10100 isolate at 0, 6, 12, 24, and 48 h. Data are presented as the mean ± standard error of three replicates. Asterisk indicates statistically significant differences from WT at each time point analyzed using Student’s *t*-test (* *p* < 0.05, ** *p* < 0.01).

The AsA content was measured in WT and *OsVTC1-1* RI1-2 line at 0, 6, 12, 24, and 48 h post inoculation (hpi) with the rice blast 10100 isolate. AsA content was significantly different between the WT and *OsVTC1-1* RI1-2 line at all time points (Figure 4). A high AsA content was observed at 6 and 12 hpi, followed by a dramatic decline at 24 hpi. AsA contents were lower in the *OsVTC1-1* RNAi line than in the WT at 0, 6, and 12 hpi, but they were higher at 24 and 48 hpi in the *OsVTC1-1* RNAi line when compared to the WT (Figure 4). These results suggest that AsA is significant during rice blast fungus infection.

### 2.5. Detection of Superoxide Anion by Nitroblue Tetrazolium (NBT) Staining and Measurement of Malondialdehyde (MDA) Content

**Figure 5:**
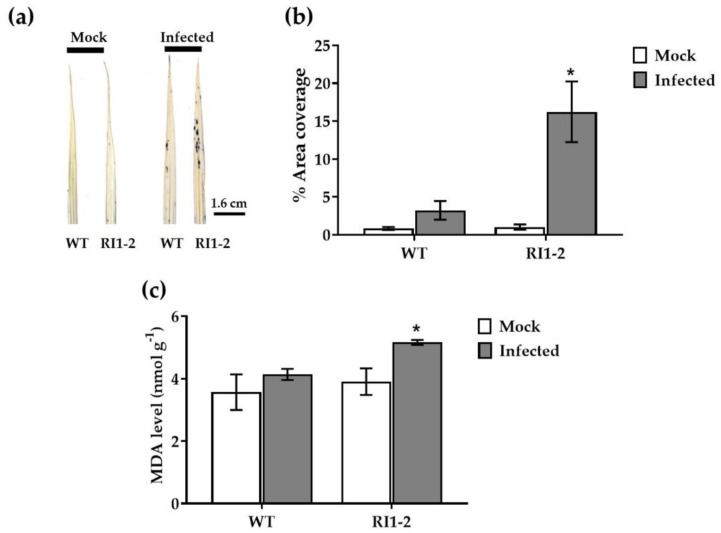
*OsVTC1-1* RNAi (RI1-2) line induces accumulation of superoxide anion and lipid peroxidation at 24 hpi with 10100 isolate. (**a**) Visualization of O_2_^−^ detected by nitroblue tetrazolium (NBT) staining; (**b**) the percentage of the leaf area covered by O_2_^−^ accumulation; (**c**) determination of lipid peroxidation by MDA measurement. Data are presented as the mean ± standard error of three replicates. Asterisk indicates statistically significant differences between mock and infected plants for each rice lines analyzed using Student’s *t*-test (* *p* < 0.05).

To investigate the mechanism of OsVTC1-1 in response to rice blast fungal infection, O_2_^−^ accumulation was measured in WT and RI1-2 leaves at 24 hpi with the 10100 isolate. The *OsVTC1-1* RNAi line accumulated more O_2_^−^ than the WT (Figure 5a,b, Table S1). A reduction in OsVTC1-1 activity leads to a higher ROS accumulation after rice blast inoculation.

The production of malondialdehyde (MDA), a byproduct of lipid peroxidation, was measured in WT and *OsVTC1-1* RI1-2 line at 24 hpi. After rice blast inoculation, there was no change in MDA content in WT leaves. Meanwhile, a significant difference was observed in MDA content between the uninfected and infected leaves of the RI1-2 line (Figure 5c). A higher level of MDA was produced in infected leaves compared to uninfected leaves. These results indicate that reducing the AsA level caused the *OsVTC1-1* RNAi line to suffer more from cell damage than WT after rice blast inoculation.

### 2.6. Transcriptome Analysis

**Figure 6:**
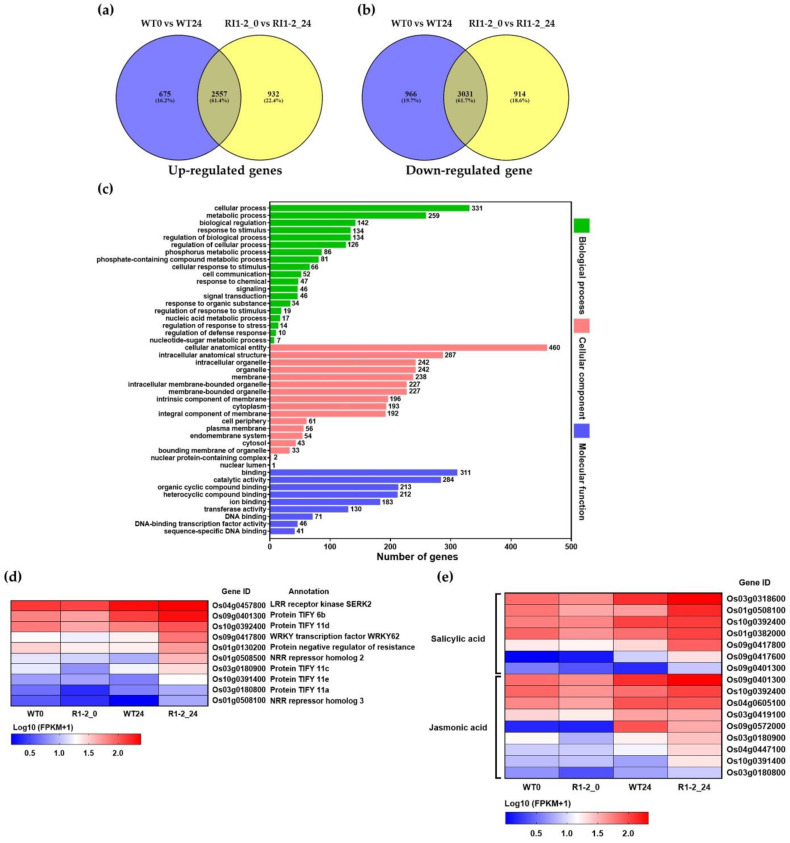
Transcriptome analysis in wild-type (WT) and *OsVTC1-1* RI1-2 line at 0 and 24 hpi with rice blast 10100 isolate. (**a**) Venn diagram of up-regulated differentially expressed genes (DEGs) and (**b**) down-regulated DEGs; (**c**) gene ontology (GO) enrichment analysis of up-regulated DEGs in the *OsVTC1-1* RI1-2 line in terms of biological process (green), cellular component (pink), and molecular function (blue); (**d**) heatmap of up-regulated DEGs related to defense response; (**e**) heatmap of up-regulated DEGs related to plant hormone synthesis and signaling pathways. The gene expression levels are presented by log10 (FPKM+1) values. Red color displays high expression levels of genes and blue color represents low expression levels of genes.

Transcriptome analysis of differentially expressed genes (DEGs) between rice-blast-inoculated WT and *OsVTC1-1* RI1-2 plants was analyzed at 0 and 24 hpi. The RNA-seq data showed that the raw sequence reads of each sample ranged from 43,787,008 to 64,022,678. Reads were filtered and mapped to a reference sequence and more than 90% of the reads aligned successfully with the reference genome (Table S2). In total, there are 9075 DEGs in WT and RI1-2 represented by 4164 up-regulated DEGs and 4911 down-regulated DEGs. Among these DEGs, 675 up-regulated DEGs and 966 down-regulated DEGs were only presented in WT, while 932 up-regulated DEGs and 914 down-regulated DEGs were only presented in RI1-2. Additionally, 2557 up-regulated DEGs and 3031 down-regulated DEGs overlapped both in WT and RI1-2 (Figure 6a,b). Principal component analysis (PCA) was performed to verify the similarity between three replicates based on the fragments per kilobase of transcript per million mapped read (FPKM) values transformed into log10 (FPKM + 1). The data of 100 common genes from the WT and *OsVTC1-1* RI1-2 line was clustered closely at 0 and 24 hpi and was grouped by time point on the PCA plot (Figure S1). The gene expression levels of 100 common genes were similar among three replicates based on log10 (FPKM + 1) values for WT and *OsVTC1-1* RI1-2 lines at 0 and 24 hpi (Figure S2, Table S3).

Table 1 lists the top 20 up- and down-regulated DEGs found only in the *OsVTC1-1* RI1-2 line ranked by log2FC value. Several defense-related genes were up-regulated at 24 hpi in the *OsVTC1-1* RI1-2 line, including *germin-like protein 8-10*, *peroxidase*, *cytochrome P450 family*, *chitinase-4 or pathogenesis-related* (*PR*)-3, *glycoside hydrolase*, *chymotrypsin protease inhibitor*, *germin-like protein 8-5*, *terpene synthase*, and *germin-like protein 8-7*. Several DEGs were down-regulated in *OsVTC1-1* RI1-2, including *S-like ribonuclease*, *tonoplast membrane integral protein*, *UDP-glucuronosyl/UDP-glucosyltransferase*, *aldo/keto reductase*, and *flavin-containing monooxygenase* (Table 1).

Gene ontology (GO) enrichment analysis was used to classify the 932 up-regulated DEGs between 0 and 24 hpi in the *OsVTC1-1* RI1-2 line (Table S4). DEGs were divided into three major categories: biological process, cellular component, and molecular function (Figure 6c). Among the three groups, DEGs were further subdivided into 19 subgroups of biological process, 17 subgroups of cellular component, and 9 subgroups of molecular function. The three most abundant biological process subgroups were cellular process (331 DEGs), metabolic process (259 DEGs), and biological regulation (142 DEGs). In addition, 10 DEGs in regulation of defense response subgroup were also enriched in biological process, including *LRR receptor kinase*, *protein TIFY*, *WRKY transcription factor WRKY62*, *protein negative regulator of resistance*, and *NRR repressor* (Figure 6d). A major subgroup of cellular component is cellular anatomical entity (460 DEGs), followed by intracellular anatomical structure (287 DEGs) and intracellular organelle (242 DEGs). In terms of molecular function, the three most significant subgroups were binding (311 DEGs), catalytic activity (284 DEGs), and organic cyclic compound binding (213 DEGs) (Figure 6c). 

An analysis of plant reactome pathways was conducted to visualize up-regulated DEGs between 0 and 24 hpi with rice blast fungus in the *OsVTC1-1* RI1-2 line. A total of 932 DEGs was identified in 82 plant reactome pathways (Table S5). The top 10 enriched pathways include metabolism and regulation; hormone signaling, transport, and metabolism; carbohydrate metabolism; growth and developmental processes; reproductive structure development; seed development; seed size regulation; amino acid metabolism; jasmonic acid signaling; and salicylic acid signaling (Figure S3a). Additionally, a number of DEGs was also assigned to enzymes involved in the synthesis and signaling of hormones, including SA and JA (Figure 6e). Moreover, DEGs involved in cellulose and phenylpropanoid synthesis were identified (Table S5).

### 2.7. Proteome Analysis

**Figure 7:**
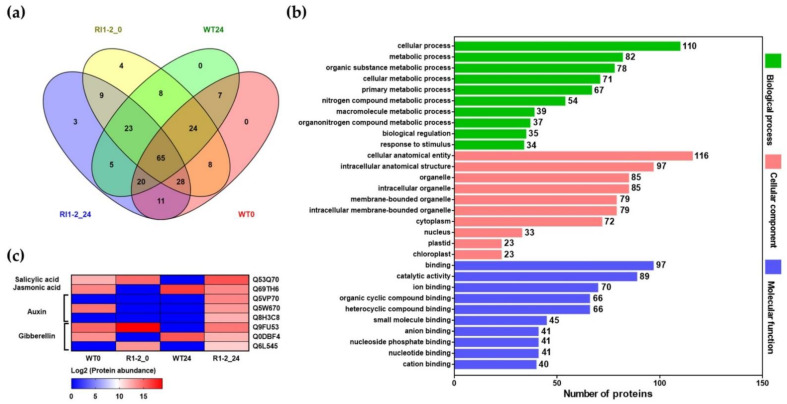
Proteome analysis in wild-type (WT) and *OsVTC1-1* RI1-2 line at 0 and 24 hpi with rice blast 10100 isolate. (**a**) Venn diagram of differentially expressed proteins (DEPs) in WT and *OsVTC1-1* RI1-2 line at 0 and 24 hpi; (**b**) gene ontology (GO) enrichment analysis of DEPs in the *OsVTC1-1* RI1-2 line at 24 hpi in terms of biological process (green), cellular component (pink), and molecular function (blue); (**c**) heatmap of DEPs related to plant hormone synthesis and signaling pathways. Red color displays high expression levels of proteins and blue color represents low expression levels of proteins.

To examine the changes in differentially expressed proteins (DEPs) after rice blast inoculation, leaf samples were collected from WT and *OsVTC1-1* RI1-2 lines at 0 and 24 hpi. A total protein extract was prepared and the peptide sequences were identified using liquid chromatography-tandem mass spectrometry (LC/MS-MS). The peptide sequence data was identified using Uniprot (http://www.uniprot.org/) (accessed 26 January 2022). Thus, 215 proteins were detected, of which 163, 152, 169, and 164 were detected in WT0, WT24, RI1-2_0, and RI1-2_24, respectively. Further, 65 proteins were common in all samples (Figure 7a). The differentially expressed proteins between the WT and *OsVTC1-1* RI1-2 line are shown in Table 2. Three proteins were specifically identified in the *OsVTC1-1* RI1-2 line, including auxin efflux carrier component 3a, probable protein phosphatase 2C 1, and IAA-amino acid hydrolase ILR1-like 8. A variety of transcription factors were found to be both increasing and decreasing in abundance in the *OsVTC1-1* RI1-2 line. There was a substantial increase in the abundance of cell-wall-related proteins, such as expansin-A2, kinesin-like protein KIN-7E, and beta-galactosidase 9, as well as defense proteins, including leucine aminopeptidase 2, endoribonuclease dicer homolog 2, and pheophorbide an oxygenase, in the *OsVTC1-1* RI1-2 line, compared to the WT after rice blast inoculation. However, glutathione reductase, superoxide dismutase, glutathione S-transferase, and 12-oxophytodienoate reductase 11 are involved in cell redox and showed a decrease in protein abundance after rice blast inoculation in the *OsVTC1-1* RI1-2 line (Table 2).

Gene ontology (GO) and plant reactome pathway analysis were used to analyze the functions and pathways of differentially expressed proteins (DEPs) in *OsVTC1-1* RI1-2 lines at 24 hpi. A list of the top 10 subgroups in three categories, comprising biological process, cellular component, and molecular function, are shown in Figure 7b and Table S6. Cellular processes, cellular anatomical entity, and binding were the most enriched subgroups in biological process, cellular component, and molecular function, respectively (Figure 7b). A total of 164 DEPs was assigned to 58 plant reactome pathways (Table S7). Metabolism and regulation; hormone signaling, transport and metabolism; growth and developmental processes; reproductive structure development; amino acid metabolism; secondary metabolism; seed development; regulation of seed size; cofactor biosynthesis; and auxin signaling were the top 10 enriched plant reactome pathways (Figure S3b). At 24 hpi, the *OsVTC1-1* RI1-2 line contained highly expressed proteins related to plant hormone synthesis and signaling pathways (Figure 7c). Proteins involved in phenylpropanoid biosynthesis were also detected in the *OsVTC1-1* RI1-2 line at 24 hpi (Table S7).

## 3. Discussion

Ascorbic acid (AsA) plays an important role in many plant processes, including cell wall biosynthesis, photosynthesis, hormone synthesis, and defense response [36]. When genes in the AsA biosynthesis pathway are modified, it affects the previously mentioned physiological pathways and mechanisms. In this study, an inclusive investigation of the *OsVTC1-1* gene in the AsA synthesis pathway and how it influenced defense responses after rice blast inoculation was conducted. In comparison with the WT, *OsVTC1-1* RI1-2 and RI1-3 lines demonstrated enhanced resistance to rice blast fungus by exhibiting fewer and smaller lesions and the induction of several defense mechanisms. Silencing *OsVTC1-1* leads to reducing the amount of AsA, an ROS-scavenging antioxidant. AsA decreases lead to an increase in ROS production, which results in HR and cell death.

The silencing of *OsVTC1-1* also impacts the abundance of non-cellulosic cell wall polysaccharides. After rice blast infection, non-cellulosic cell wall polysaccharides increased in the *OsVTC1-1* RI1-2 line. These changes could influence the ability of the fungus to penetrate the cell wall.

Photosynthesis parameters alter as a result of pathogen infection [37]. This study revealed that the maximum efficiency of photosystem II (PSII), measured as F_v_/F_m_, was reduced in the infected leaves of the WT but not in the *OsVTC1-1* RI1-2 line. The reduction in *OsVTC1-1* gene expression protected photosynthetic efficiency in *OsVTC1-1* RNAi lines during *M. oryzae* infection. Moreover, the amount of AsA decreased 24 h after rice blast inoculation in both WT and *OsVTC1-1* RI1-2 lines. These results are consistent with a previous report, in which AsA contents decreased in infected leaves and roots at 24 h after infection with *Phytophthora cinnamomi* [38]. Additionally, it was found that the *OsVTC1-1* RI1-2 line accumulates more O_2_^−^ and MDA than the WT. O_2_^−^ is an ROS that is essential in initiating signaling pathways and defense-related gene activation, as well as interacting with proteins, DNA, and lipids to cause cell damage [39]. MDA is a byproduct of lipid peroxidation caused by ROS. Increased lipid peroxidation was observed in HR cell death after *M. oryzae* infection in rice [32].

*OsVTC1-1* gene silencing enhances resistance to *M. oryzae* by disturbing gene and protein expression in multiple pathways, including defense response and hormone signaling. DEGs and DEPs between WT and *OsVTC1-1* RI1-2 line at 0 and 24 hpi were identified. Among the up-regulated DEGs associated with defense response, determined in this study and reported in previous studies, include germin-like protein family (GLP), peroxidase, and pathogenesis-related (PR) proteins. GLPs play an essential role in defense against *Aspergillus flavus* [40]. Peroxidases are associated with ROS generation and interacting with plant defense molecules, such as salicylic acid (SA), chitooligosaccharides (COSs), and aromatic monoamines (AMAs) [41]. Additionally, the RNA-seq data revealed that the *peroxidase* gene was expressed at the highest level at 12 hpi with rice blast fungus in Nipponbare [42]. PR proteins are generated during plant–pathogen interactions, which can inhibit the growth of pathogens [43]. A previous study reported that the expression of the *PR-3* (At2g43590) gene was induced at 6 and 24 h after inoculation with *Alternaria brassicae* [44]. Moreover, in this study, through proteome analysis, DEPs related to cell wall biosynthesis and defense response were identified, including expansin, which is involved in plant cell wall expansion [45]; kinesin, which is necessary in cellulose microfibril and microtubule organization, signal transduction, and vesicle transport [46]; and leucine aminopeptidase, which acts downstream of JA synthesis and is involved in defenses against herbivores by developing late wounds in tomato [47].

Hormone signaling pathways are also disrupted by *OsVTC1-1* silencing after rice blast inoculation. Particularly, SA and JA were the most effected plant hormones during infection. SA acts as signaling molecules in systemic acquired resistance (SAR) [48]. Consistent with a previous report, genes involved in hormone signaling molecules (SA and JA) were detected through transcriptome analysis during rice–rice blast interaction [49]. The SA activates the immune response against biotrophic pathogens, whereas the JA activates the immune response against necrotrophic pathogens [50]. SA and JA are, thus, required for rice defense signals against *M*. *oryzae*, a hemibiotrophic fungal pathogen. These results suggest that several defense mechanisms are induced in response to rice blast infection, especially plant hormone synthesis and signaling pathways.

## 4. Materials and Methods

### 4.1. Plant Materials and Growth Conditions

Transgenic rice seeds with knocked-down expression of the *OsVTC1-1* gene (RI1-2 and RI1-3) and Zhonghua17 (ZH17) background of RNAi plants or WT were acquired from the Biotechnology Research Institute, Chinese Academy of Agricultural Sciences, Beijing, China [18]. Rice seeds were cultivated as previously described [35]. Briefly, rice seeds were germinated in a Petri dish containing moist tissue papers for 4–5 days, grown in plastic pots (9 cm diameter) containing soil (eight seeds per pot) and cultured at 24 °C under photoperiod cycle of 12 h light/dark and 90% humidity in a plant growth chamber (Reftech BV, Sassenheim, The Netherlands). Three-week-old rice seedlings were used for rice blast fungus inoculation in cell wall monosaccharide composition analysis and photosynthetic efficiency analysis.

Due to insufficient amount of *OsVTC1-1* RI line seeds, only *OsVTC1-1* RI1-2 line was used in AsA content measurement, detection of superoxide anion accumulation, MDA content measurement, and transcriptome and proteome analysis. WT and RI1-2 rice seeds were cultured in a greenhouse at 30 °C with 80% humidity and 12 h photoperiod. Khao Dawk Mali 105 (KDML105) and Jao Hom Nin (JHN), Thai rice varieties, were used as susceptible and resistant varieties to rice blast fungus, respectively. Three-week-old rice seedlings were used for rice blast fungus inoculation.

### 4.2. Fungal Materials and Culture Conditions

Rice blast fungus Guy11 isolate, which can infect the ZH17 rice variety, was used for rice inoculation in cell wall monosaccharide composition analysis and photosynthetic efficiency analysis. Guy11 is stored in Nicholas J. Talbot’s laboratory at the School of Biosciences, University of Exeter, UK. Fungal isolates in filter paper were cultured on solid complete medium (CM) and incubated at 24 °C with a 12-h shift of the light–dark cycle in a fungal growth room for 5 days. The fungal plate was sub-cultured by cutting a 0.5 cm × 0.5 cm mycelia piece to new medium and incubating it using the above conditions for 5 days before inoculation [51].

Rice blast fungus 10100 isolate that causes the disease in the ZH17 rice variety was collected from Srakaew, Thailand, in 2006. It was used for rice inoculation samples analyzed for AsA content, detection of superoxide anion accumulation, MDA content, and transcriptome and proteome analysis. The filter paper stock of 10100 isolate was cultured on rice flour agar (RFA; 20 g of rice powder, 28 g of agar and 2 g of yeast extract in 1 L of distilled water) in a Petri dish and incubated at 28 °C for 7 days. Then, the fungal plate was sub-cultured by cutting a 0.5 cm × 0.5 cm mycelia piece to new RFA medium and incubating the new piece as previously described. Fungal mycelia were scraped off using a stainless-steel spatula to induce sporulation and placed under UV light for 3 days before inoculation.

### 4.3. Rice Inoculation and Pathogenicity assay

Conidia of Guy11 were collected from culture plates by washing with sterile water and filtered using sterile Miracloth (Calbiochem, San Diego, CA, USA). Conidia suspensions were centrifuged at 8000× *g* for 5 min at room temperature. Conidia suspensions were mixed with 0.2% (*w*/*v*) gelatin and adjusted to 1 × 10^5^ spores mL^−1^. Conidia suspensions were used immediately after preparation to avoid germination. Conidia suspensions were sprayed on 3-week-old WT and *OsVTC1-1* RNAi rice seedlings (5 mL per three pots) with an artist’s airbrush (Badger Airbrush, Franklin Park, IL, USA). For mock inoculations, plants were sprayed with 0.2% (*w*/*v*) gelatin only. The inoculated plants were kept in closed containers for 24 h and, afterwards were stored in a plant-growth-controlled environment room at 24 °C with a photoperiod cycle of 12 h light/dark and 90% humidity. Infected leaves were collected 4 days after inoculation, immediately immersed in the liquid nitrogen, and stored at −80 °C until cell wall extraction. Rice blast symptoms were examined at 7 days post inoculation. Furthermore, conidia suspensions were also used for a spot inoculation method. Twenty microliter droplets of conidia suspension were inoculated on the adaxial surfaces of detached WT and *OsVTC1-1* RNAi leaves at three drops per leaf. Inoculated rice leaves were incubated in moist chambers for 5 days for photosynthetic efficiency analysis.

For 10100 isolates, conidia were harvested by washing them out from the RFA medium with sterile water. To make conidia suspensions, conidia were mixed with 0.3% (*w*/*v*) gelatin and 0.01% (*v*/*v*) Tween 20 and adjusted to 1 × 10^5^ spores mL^−1^. Conidia suspensions were sprayed to 3-week-old WT and RI1-2 rice plants using nano-sprayer machine. Five mL of inoculum was applied per three pots. For mock inoculations, rice seedlings were sprayed with 0.3% (*w*/*v*) gelatin and 0.01% (*v*/*v*) Tween 20. Inoculated rice plants were kept in the dark at 25 °C and 80–100% humidity overnight and transferred to the greenhouse. Leaf samples were collected at 0, 6, 12, 24, and 48 h after inoculation for AsA content measurement and were collected at 0 and 24 h for detection of superoxide anion, measurement of MDA content, and transcriptome and proteome analysis. Moreover, disease symptoms were also observed at 7 days post inoculation.

### 4.4. Cell Wall Sugar Composition Analysis

For cell wall preparation, GC-MS analysis and data analysis were performed as previously reported [35]. Briefly, 3-week-old leaves of WT and *OsVTC1-1* RNAi lines, both mock and rice blast fungus treated, were collected at 4 days after inoculation and stored in liquid nitrogen. Frozen leaf samples were ground with a TissueLyser (Qiagen, Hilden, Germany) twice for 30 s at 30 Hz frequency. The alcohol-insoluble residue (AIR) preparation method was used for cell wall preparation as previously described [52]. Monosaccharide compositions of cell wall samples were investigated using an Agilent 7200 series-accurate mass Q-TOF GC-MS together with a 7890A GC system (Agilent Technologies, Santa Clara, CA, USA). Data analysis was performed using Agilent technologies MassHunter qualitative version B.07.00 and quantitative software version B.08.00 (Agilent Technologies, Santa Clara, CA, USA). Data from mock inoculation were previously used and published in Lamanchai et al. [35]

### 4.5. Photosynthetic Efficiency Analysis

Photosynthetic efficiency in WT and *OsVTC1-1* RNAi line after rice blast inoculation was determined by measurement of chlorophyll fluorescence FluorImager software version 2.2 (CF Imager, Technologica Ltd., Colchester, UK). Detached leaves were placed in the CF Imager and dark adapted for 20 min before measurement of basal fluorescence (F_0_). Maximal fluorescence (F_m_) was induced by a saturating flash of blue light (0.5 s at 6000 µmol m^−2^ s^−1^). Variable fluorescence (F_v_) was calculated as F_m_-F_0_ to provide the dark-adapted quantum efficiency of photosystem II (F_v_/F_m_) [53]. Three replicates were performed for each rice line.

### 4.6. Measurement of Ascorbic Acid Content

To measure AsA content in WT and RI1-2 line, leaf samples were collected at 0, 6, 12, 24, and 48 h after inoculation and stored in liquid nitrogen. AsA content measurement with three biological replicates was performed as previously described [54] with slight modification according to Lamanchai et al. [35]. Finally, the absorbance was measured at 525 nm using a spectrophotometer and the concentrations were calculated using a standard calibration curve.

### 4.7. Detection of Superoxide Anion by Nitroblue Tetrazolium (NBT) Staining

Superoxide anion (O_2_^−^), one of the most important ROS in cells, was measured by NBT staining following methodology described by Kumar et al. [55]. Leaf samples were collected at 24 h post inoculation and immersed in 50 mM sodium phosphate buffer (pH 7.5) containing 0.2% (*w*/*v*) NBT for detection of O_2_^−^. Samples were incubated in the dark at room temperature overnight. After the NBT was drained off, chlorophyll was removed by immersing samples in absolute ethanol and heating in a water bath at 80 °C until the green color was completely removed. The samples were transferred to a paper towel saturated with 60% (*v*/*v*) glycerol and stained samples were photographed. O_2_^−^ was detected as a dark blue color. The percentage area covered by O_2_^−^ accumulation was measured using ImageJ software version 1.53k (Wayne Rasband, National Institutes of Health, Bethesda, MD, USA). Three replicates were performed for each rice line.

### 4.8. Measurement of Malondialdehyde (MDA) Content

To measure MDA content, 0.1 g of leaf samples was used for extraction in 1 mL of 0.1% (*w*/*v*) TCA for 10 min and centrifuged at 10,000× *g* at 4 °C for 15 min. Seven hundred microliters of supernatant was transferred to new microcentrifuge tubes. One milliliter of 0.5% (*v*/*v*) tertiary butyl alcohol (TBA) was added to supernatant, boiled at 95 °C for 25 min, and quickly cooled on ice for 5 min. Absorbance was measured using spectrophotometer at 532 and 600 nm. MDA content was calculated based on calculations in Health and Packer 1968 [56]. Three replicates were performed for each experiment.

### 4.9. Transcriptome Analysis

Leaf samples of WT and *OsVTC1-1* RI1-2 line were collected at 0 and 24 hpi and labelled as W0, W24, RI1-2_0, and RI1-2_24, respectively. Total RNA was extracted using the GF-1 Total RNA extraction kit (Vivantis, Shah Alam, Malaysia) according to the manufacturer’s instructions. Pair-end sequencing using Illumina Novaseq 6000 platform (Illumina, San Diego, CA, USA) was conducted by Novogene Bioinformatics Technology Co., Ltd., Beijing, China. For data analysis, the RNA-seq data was performed as previously described [35] using apps in the CyVerse Discovery Environment (DE) [57]. The sequences were mapped using IRGSP-1.0 as the reference genome sequence. DEGs were identified using adjusted *p*-value < 0.05 and |log2FC| > 1 as cutoff criterion. The Rice Annotation Project Database (RAP-DB, http://rapdb.dna.affrc.go.jp/) was used to find gene descriptions for DEGs (accessed on 11 June 2022) [58,59]. DEGs with up- and down-regulated genes at 0 and 24 h after rice blast inoculation between WT and *OsVTC1-1* RI1-2 line were compared. A Venn diagram was constructed using Venny version 2.1.0. (https://bioinfogp.cnb.csic.es/tools/venny/index.html, accessed on 8 June 2022). Gene ontology (GO, http://geneontology.org/) enrichment analysis was performed to classify the function of DEGs (accessed on 11 June 2022) [60,61,62]. Pathway analysis was analyzed using plant reactome database (http://plantreactome.gramene.org/, accessed on 11 June 2022) [63]. A heatmap of up-regulated DEGs and correlation between three biological replicates was generated based on the fragments per kilobase of transcript per million mapped reads (FPKM) values transformed by log10 (FPKM+1) using GraphPad Prism version 9.0.0 for Windows (San Diego, CA, USA). Principal component analysis (PCA) was carried out with factoextra package in RStudio [64] using a log10 transformation of (FPKM+1) values to compare similarity between three biological replicates.

### 4.10. Proteome Analysis

Protein extraction, digestion, LC-MS/MS determination, and protein identification were achieved as previously described [35]. Briefly, leaf samples were ground into a powder with liquid nitrogen. Protein was extracted using 0.5% (*w*/*v*) sodium dodecyl sulfate (SDS) and the concentration was measured by the method of Lowry [65]. Protein samples were digested in gel with trypsin (1:20 ratio) (Promega, Madison, WI, USA). The tryptic peptide samples were prepared for injection into an Ultimate3000 Nano/Capillary LC System (Thermo Scientific, Waltham, MA, USA) coupled to a Hybrid quadrupole Q-Tof impact II™ (Bruker Daltonics, Bremen, Germany) equipped with a Nano-captive spray ion source. MaxQuant version 1.6.6.0 (Max-Planck Institute for Biochemistry, Planegg, Germany) was used to quantify the peptide MS signal intensities from the analyzed MS/MS data from LC-MS [66]. For protein identification, the data were searched against the Uniprot database (released date 26 January 2022). A Venn diagram was constructed using Venny version 2.1.0. (https://bioinfogp.cnb.csic.es/tools/venny/index.html, accessed on 8 June 2022). Gene ontology (GO, http://geneontology.org/) enrichment analysis was performed to classify the function of proteins (accessed on 11 June 2022) [60,61,62]. Pathway analysis was analyzed using plant reactome database (http://plantreactome.gramene.org/, accessed on 11 June 2022) [63]. A heatmap of identified proteins was created based on log2 (protein abundance) value using GraphPad Prism version 9.0.0 for Windows (San Diego, CA, USA).

### 4.11. Statistical Analyses

The data were reported as mean ± standard error (SE) and were analyzed using IBM SPSS Statistics v. 26 software (IBM, New York, NY, USA). A Student’s *t*-test was performed to determine statistical differences at a significant level of 0.05.

## 5. Conclusions

In this study, we report that the *OsVTC1-1* gene is involved in the interaction between rice and rice blast fungus. Disease reaction, cell wall monosaccharide composition, photosynthetic efficiency, AsA content, O_2_^−^ accumulation, and MDA content were analyzed in WT and *OsVTC1-1* RNAi lines after rice blast inoculation. An enhanced rice blast resistance was observed in the *OsVTC1-1* RNAi lines. A significant difference between *OsVTC1-1* RNAi lines and WT was observed in the composition of monosaccharides on cell walls, photosynthetic efficiency, O_2_^−^ accumulation, and levels of MDA. Transcriptome and proteome analysis revealed that rice blast inoculation induced defense-related genes and proteins. Pathways involved in the biosynthesis of plant hormones, especially SA and JA, were identified in the *OsVTC1-1* RNAi line after rice blast inoculation. Our results indicate that a reduction in the expression of the *OsVTC1-1* gene in the AsA synthesis pathway enhances resistance to rice blast fungus via multiple defense mechanisms.

## Figures and Tables

**Table 1:** Top 20 up- and down-regulated differentially expressed genes (DEGs) in *OsVTC1-1* RI1-2 line at 24 h after inoculation with rice blast 10100 isolate. Genes were ranked by log2FC value.

**Up-Regulated DEGs of RI1-2**			
**No.**	**Gene ID**	**Gene Description**	**Log2FC**
1	Os08g0189900	Germin-like protein 8-10, disease resistance	10.156
2	Os12g0503000	Allantoin transporter	8.199
3	Os03g0363100	Armadillo-like helical domain containing protein	8.186
4	Os06g0522300	Haem peroxidase family protein	8.156
5	Os05g0473101	Hypothetical gene	7.671
6	Os07g0541900	Similar to KI domain interacting kinase 1	7.659
7	Os07g0677200	Peroxidase, negative regulation of rice resistance to blast disease	7.640
8	Os08g0507100	Cytochrome P450 family protein	7.599
9	Os04g0493400	Chitinase-4, Pathogenesis-related (PR)-3	7.596
10	Os07g0539100	Glycoside hydrolase family 17 protein	7.476
11	Os06g0244000	Similar to anthranilic acid methyltransferase	7.395
12	Os01g0731150	Hypothetical protein	7.374
13	Os09g0447300	Cytochrome P450 family protein	7.297
14	Os01g0615100	Chymotrypsin protease inhibitor	7.297
15	Os01g0615050	Chymotrypsin inhibitor	7.274
16	Os09g0400400	Cinnamyl alcohol dehydrogenase	7.260
17	Os08g0189400	Germin-like protein 8-5, disease resistance	7.104
18	Os09g0319800	Terpene synthase-like domain containing protein	7.067
19	Os11g0618700	Protein of unknown function DUF594 domain containing protein.	7.001
20	Os08g0189600	Germin-like protein 8-7, disease resistance	6.956
**Down-Regulated DEGs of RI1-2**			
**No.**	**Gene ID**	**Gene Description**	**Log2FC**
1	Os09g0537700	S-like ribonuclease, regulation of photomorphogenesis	−9.326
2	Os05g0579600	Homeodomain-like containing protein.	−8.560
3	Os01g0975900	Similar to tonoplast membrane integral protein ZmTIP1-2	−7.913
4	Os01g0922700	Conserved hypothetical protein.	−7.627
5	Os05g0409500	Similar to MtN21 protein.	−7.565
6	Os01g0973100	Hypothetical conserved gene	−7.496
7	Os06g0107100	Protein of unknown function DUF819 family protein	−7.267
8	Os07g0628900	Similar to KI domain interacting kinase 1	−7.125
9	Os01g0734600	UDP-glucuronosyl/UDP-glucosyltransferase family protein	−6.893
10	Os03g0371300	Similar to Cytochrome P450	−6.689
11	Os12g0493900	Armadillo-like helical domain containing protein.	−6.654
12	Os07g0142900	Aldo/keto reductase family protein.	−6.645
13	Os04g0282400	Flowering-promoting factor 1-like protein 4	−6.383
14	Os05g0500101	Hypothetical gene	−6.379
15	Os07g0111900	Flavin-containing monooxygenase FMO family protein	−6.326
16	Os07g0558300	Inositol monophosphatase family protein	−6.267
17	Os03g0223100	Cytochrome P450 family protein	−6.242
18	Os08g0249900	Similar to Gibberellin 20 oxidase 2	−6.239
19	Os01g0733500	Similar to dehydration-induced protein RD22-like protein 1	−6.185
20	Os04g0583900	MYB-related transcription factor	−6.183

**Table 2:** Differentially expressed proteins in wild-type (WT) and *OsVTC1-1* RI1-2 line at 0 and 24 h post inoculation with 10100 isolate.

No.	Protein ID	Protein Name	Log2 Protein Abundance			
			WT0	RI1-2_0	WT24	RI1-2_24
1	Q5VP70	Auxin efflux carrier component 3a	0	0.00	0	13.89
2	Q942P9	Probable protein phosphatase 2C 1	0	0.00	0	13.40
3	Q8H3C8	IAA-amino acid hydrolase ILR1-like 8	0	0.00	0	12.32
4	Q75KV9	Transcription factor BHLH148	14.86	0.00	0.00	12.16
5	Q53Q70	Transcription factor TGAL4	12.27	14.91	0.00	15.84
6	Q9FWX2	NAC domain-containing protein 7	13.61	13.03	0.00	15.28
7	Q9FY82	NAC domain-containing protein 82	14.16	17.64	11.13	13.48
8	Q6K537	Dof zinc finger protein 3	0.00	15.55	12.77	11.53
9	A4LBC0	B3 domain-containing protein LFL1	12.15	17.90	16.00	0.00
10	Q7Y0V7	Homeobox-leucine zipper protein ROC6	12.86	16.98	15.16	0.00
11	Q6ZBS8	Transcription factor TGAL10	0.00	12.66	12.05	0.00
12	Q40636	Expansin-A2	14.33	0.00	0.00	13.32
13	B9FFA3	Kinesin-like protein KIN-7E	15.97	14.95	15.44	17.06
14	Q5Z7L0	Beta-galactosidase 9	13.81	0.00	12.71	13.43
15	Q7XPY5	Putative beta-glucosidase 15	11.39	18.11	10.45	15.45
16	Q6K669	Leucine aminopeptidase 2	0.00	12.06	15.04	17.61
17	Q3EBC8	Endoribonuclease dicer homolog 2	13.73	18.60	14.93	16.27
18	Q0DV66	Pheophorbide a oxygenase	15.81	0.00	14.65	16.10
19	Q0D5R3	Cysteine-rich receptor-like protein kinase 6	14.21	19.46	0.00	14.71
20	Q6Z8P4	Plant intracellular Ras-group-related LRR protein 4	14.85	15.68	13.29	14.80
21	Q7X7X4	Cytochrome P450	13.27	17.21	0.00	13.74
22	P48642	Glutathione reductase	15.75	15.39	15.06	0.00
23	P93407	Superoxide dismutase	0.00	14.93	12.14	11.05
24	Q65XA0	Probable glutathione S-transferase DHAR1	0.00	13.86	16.46	11.51
25	B9FSC8	Putative 12-oxophytodienoate reductase 11	13.27	15.73	12.51	0.00

## Data Availability

Not applicable.

## Supplementary Materials

The following are available online at https://www.mdpi.com/article/10.3390/plants11172189/s1, Figure S1: Principal component analysis (PCA) plot of log10 (FPKM+1) values of 100 common genes from WT and *OsVTC1-1* RNAi lines at 0 and 24 h after rice blast inoculation between three replicates; Figure S2: Heatmap of the expression levels of 100 common genes from WT and *OsVTC1-1* RI1-2 line at 0 and 24 hpi among three replicates. The color scale on the right represents the fragments per kilobase of transcript per million mapped reads (FPKM) values transformed by log10 (FPKM+1). Red and blue represent high- and low-expression levels of genes, respectively; Figure S3: Plant reactome pathway enrichment analysis. (a) The top 10 enriched plant reactome pathways of DEGs in the *OsVTC1-1* RI1-2 line between 0 and 24 hpi; (b) The top 10 enriched plant reactome pathways of DEPs in the *OsVTC1-1* RI1-2 line at 24 hpi; Table S1: Quantification of O^2–^ accumulation in a mock-inoculated and rice-blast-infected leaves of WT and *OsVTC1-1* RI1-2 line using ImageJ software; Table S2: Summary of RNA-seq data and reads mapping; Table S3: FPKM expression values represented as log10 (FPKM+1) values of 100 common genes from WT and *OsVTC1-1* RI1-2 lines with three replicates at 0 and 24 hpi; Table S4: Gene ontology (GO) enrichment analysis of up-regulated DEGs in the *OsVTC1-1* RI1-2 line between 0 and 24 hpi. The GO categories include biological process (BP), cellular component (CC), and molecular function (MF); Table S5: Plant reactome pathway analysis of up-regulated DEGs in the *OsVTC1-1* RI1-2 line between 0 and 24 hpi; Table S6: The top 10 gene ontology (GO) enrichment analysis of DEPs in the *OsVTC1-1* RI1-2 line at 24 hpi. The GO categories include biological process (BP), cellular component (CC), and molecular function (MF); Table S7: Plant reactome pathway analysis of DEPs in the *OsVTC1-1* RI1-2 line at 24 hpi.
