# Supplementary material for: OsVTC1-1 Gene Silencing Promotes a Defense Response in Rice and Enhances Resistance to Magnaporthe oryzae
Source: Plants (Basel). 2022 Aug 24;11(17):2189. doi: 10.3390/plants11172189 (PMC9460107; doi:10.3390/plants11172189)
Supplement: Supplementary file 1 [file plants-11-02189-s001.zip › plants-1838402-supplementary.pdf]

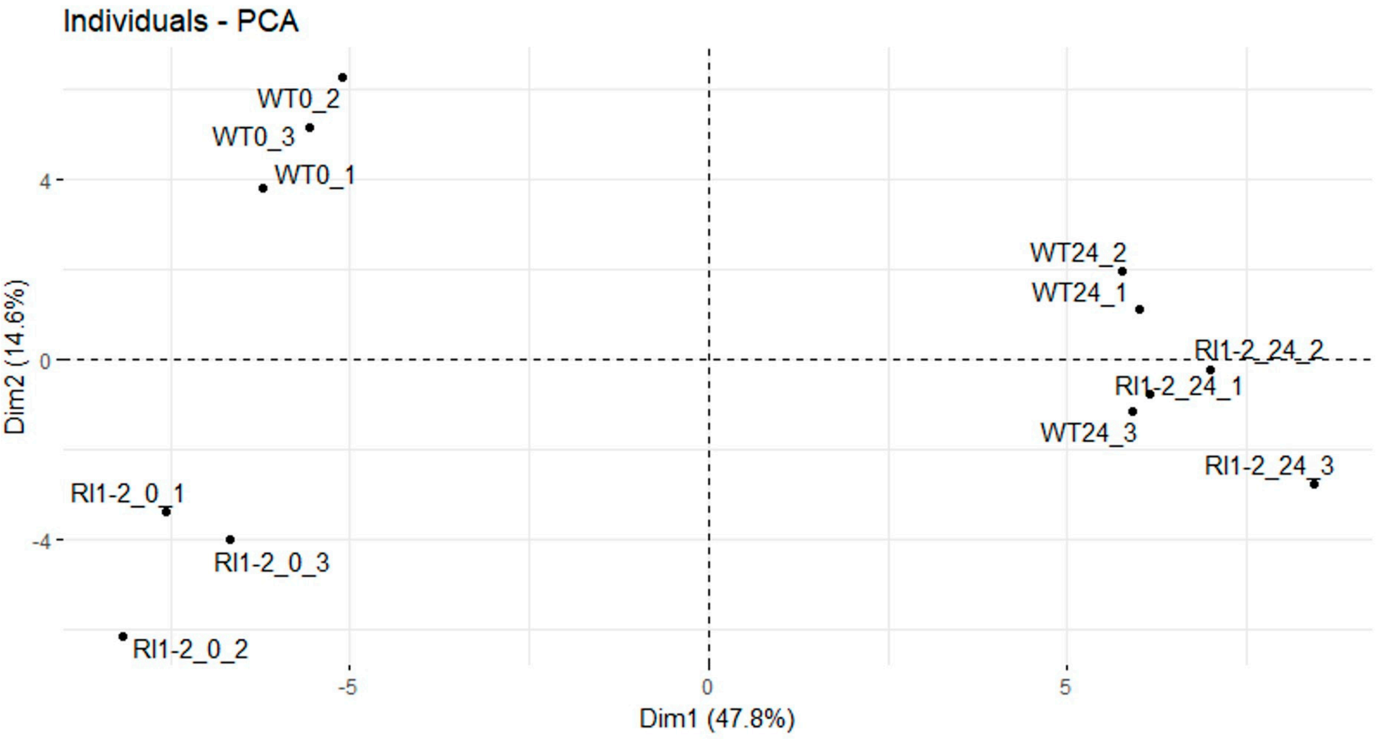

Figure S1. Principal component analysis (PCA) plot of log10 (FPKM+1) values of 100 common genes from wild type (WT) and *OsVTC1-1* RNAi lines at 0 and 24 h after rice blast inoculation between three replicates.

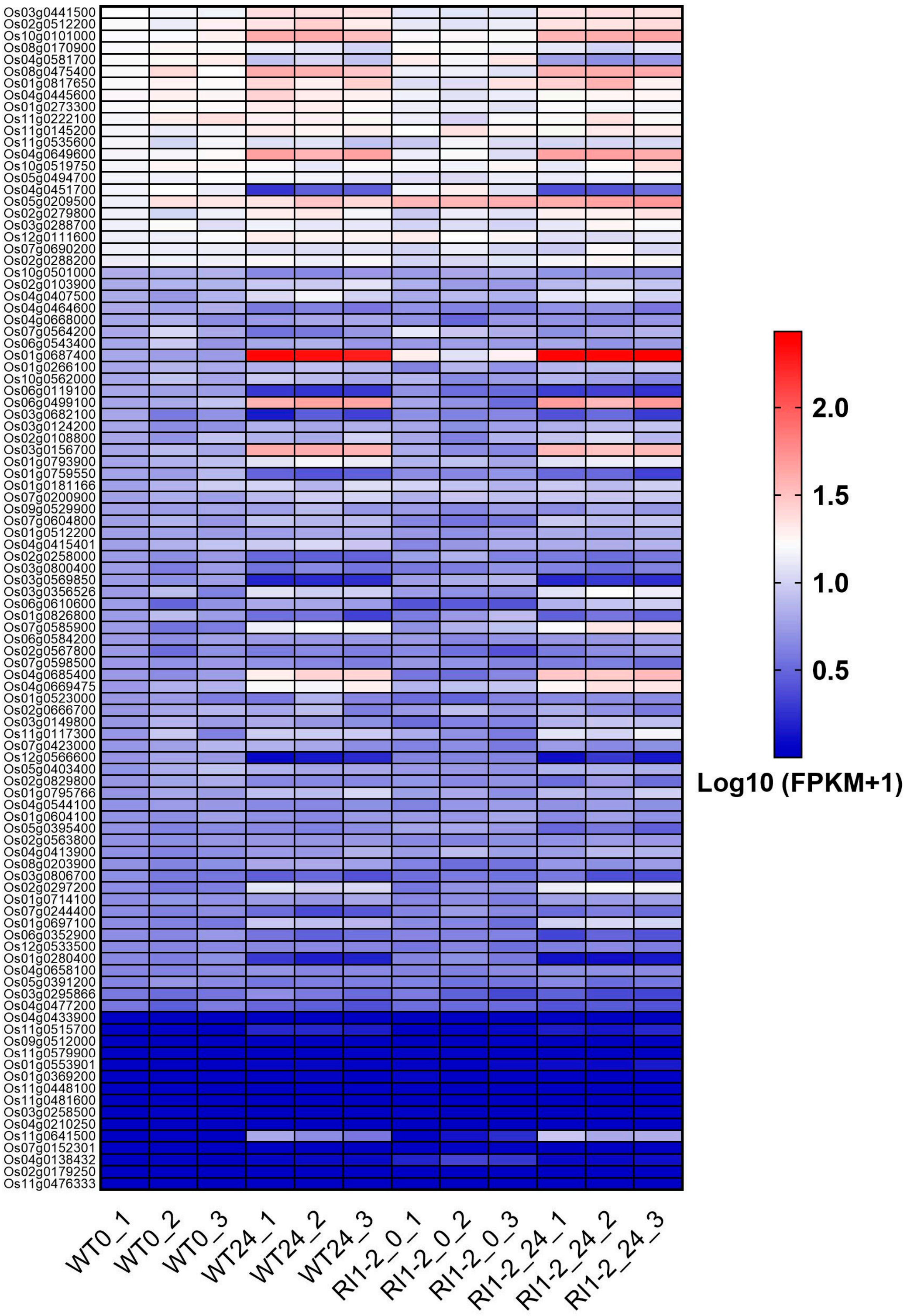

Figure S2. Heatmap of the expression levels of 100 common genes from wild type (WT) and *OsVTC1-1* RI1-2 line at 0 and 24 h after rice blast inoculation among three replicates. The color scale on the right represents the fragments per kilobase of transcript per million mapped reads (FPKM) values transformed by log10 (FPKM+1). Red and blue represent high and low expression levels of genes, respectively.

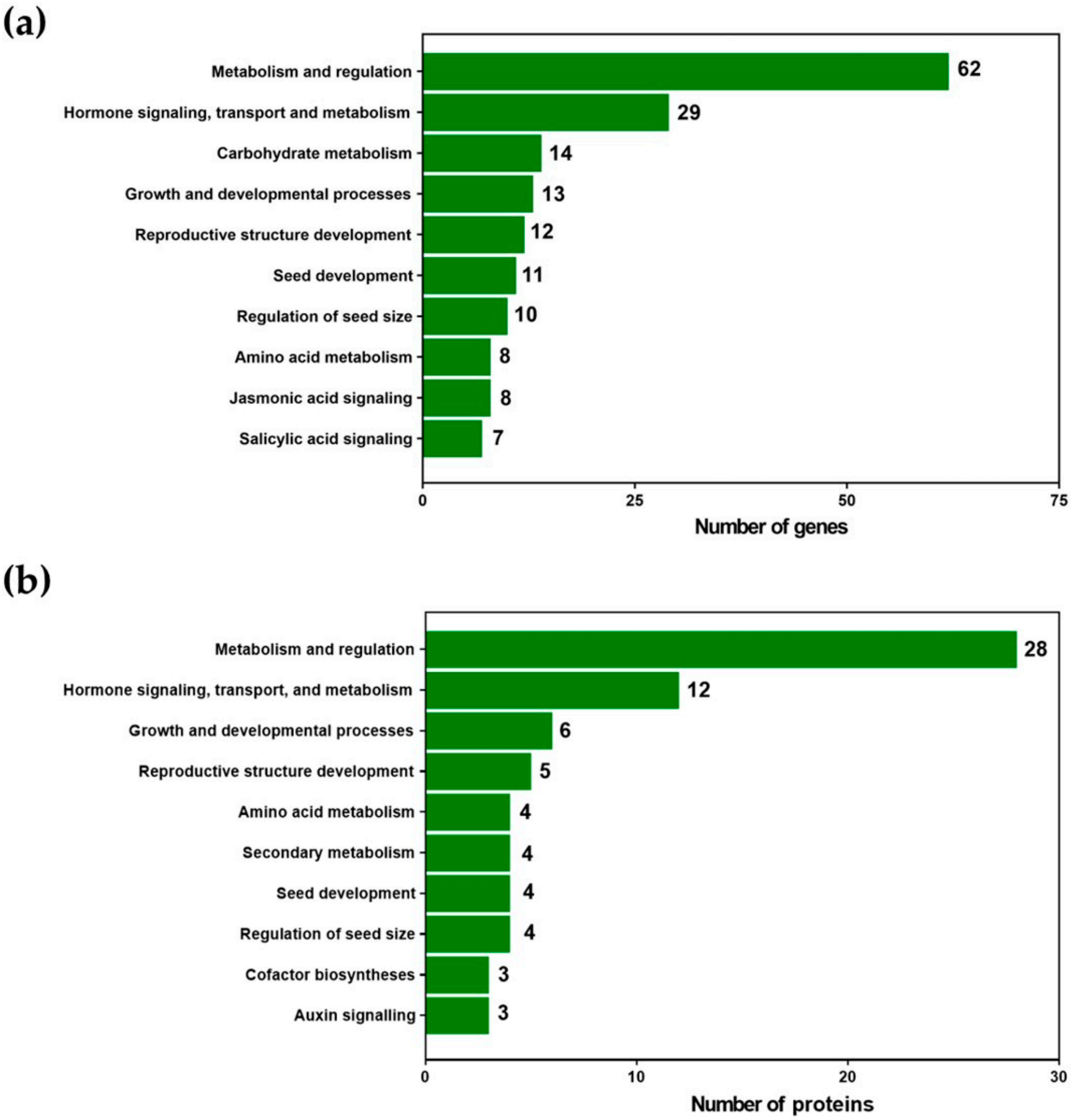

Figure S3. Plant reactome pathway enrichment analysis. (**a**) Top 10 enriched plant reactome pathway of differentially expressed genes (DEGs) in *OsVTC1-1* RI1-2 line between 0 and 24 h after inoculation; (**b**) Top 10 enriched plant reactome pathway of differentially expressed proteins (DEPs) in *OsVTC1-1* RI1-2 line at 24 h after inoculation.

Table S1. Quantification of O_2^- accumulation in mock-inoculated and rice blast-infected leaves of wild type and *OsVTC1-1* RI1-2 line using ImageJ software.

Sample names	Total area (pixel)	O_2^- detected area (pixel)	% Area coverage
WT_1_mock	10545	114	1.08
WT_2_mock	14433	66	0.46
WT_3_mock	11197	106	0.95
RI1-2_1_mock	10490	171	1.63
RI1-2_2_mock	17389	179	1.03
RI1-2_3_mock	17174	69	0.40
WT_1_infected	15769	836	5.30
WT_2_infected	14484	152	1.05
WT_3_ infected	13093	433	3.31
RI1-2_1_ infected	15571	1860	11.95
RI1-2_2_ infected	13187	1650	12.51
RI1-2_3_ infected	11133	2698	24.23

Table S2. Summary of RNA-seq data and reads mapping.

Sample names	Raw reads	Clean reads	Mapped Reads (%)	Q30 (%)	%GC
WT0_1	49,358,868	24,188,921	21,924,616 (93.9%)	92.97	52.17
WT0_2	50,914,470	25,005,334	22,729,345 (94.0%)	93.30	51.98
WT0_3	50,088,688	24,584,995	22,338,384 (94.1%)	93.01	52.11
WT24_1	45,422,594	22,269,915	20,161,089 (93.8%)	93.07	51.53
WT24_2	44,683,164	21,879,781	19,672,203 (93.2%)	92.76	51.51
WT24_3	49,808,422	24,439,085	22,100,860 (93.5%)	93.17	51.04
RI1-2_0_1	47,677,020	23,306,813	20,656,280 (91.9%)	92.44	51.87
RI1-2_0_2	44,965,952	22,057,704	19,924,245 (93.4%)	93.00	51.11
RI1-2_0_3	43,787,008	21,491,078	19,534,587 (94.1%)	93.09	52.16
RI1-2_24_1	64,022,678	31,552,045	28,495,504 (93.4%)	93.56	51.48
RI1-2_24_2	57,001,548	28,076,885	25,560,794 (94.2%)	93.59	52.10
RI1-2_24_3	44,690,738	21,910,953	19,662,846 (93.1%)	92.95	51.70

Table S3. FPKM expression values represented as log10 (FPKM+1) values of 100 common genes from wild type (WT) and *OsVTC1-1* RI1-2 lines with three replicates at 0 and 24 h after rice blast inoculation.

No.	Gene ID	Sample names											
		WT0_1	WT0_2	WT0_3	WT24_1	WT24_2	WT24_3	RI1-2_0_1	RI1-2_0_2	RI1-2_0_3	RI1-2_24_1	RI1-2_24_2	RI1-2_24_3
1	Os03g0441500	1.214	1.177	1.159	1.364	1.351	1.355	1.101	1.093	1.090	1.330	1.368	1.351
2	Os02g0512200	1.207	1.121	1.270	1.333	1.436	1.300	1.097	1.076	1.134	1.349	1.344	1.376
3	Os10g0101000	1.205	1.202	1.272	1.609	1.611	1.511	1.187	1.183	1.228	1.554	1.608	1.636
4	Os08g0170900	1.202	1.242	1.201	1.168	1.101	1.011	1.222	1.199	1.177	1.107	1.012	1.124
5	Os04g0581700	1.199	1.238	1.288	0.936	1.028	0.937	1.294	1.182	1.329	0.772	0.686	0.722
6	Os08g0475400	1.198	1.379	1.211	1.615	1.591	1.478	1.144	1.143	1.077	1.578	1.611	1.618
7	Os01g0817650	1.192	1.260	1.221	1.397	1.270	1.427	1.058	1.060	1.346	1.423	1.576	1.276
8	Os04g0445600	1.191	1.271	1.247	1.423	1.307	1.280	1.163	1.080	1.120	1.236	1.255	1.249
9	Os01g0273300	1.184	1.227	1.224	1.297	1.296	1.221	1.133	1.129	1.078	1.195	1.169	1.171
10	Os11g0222100	1.180	1.284	1.351	1.271	1.268	1.238	1.142	1.021	1.189	1.232	1.352	1.230
11	Os11g0145200	1.174	1.136	1.173	1.305	1.240	1.281	1.212	1.340	1.258	1.239	1.309	1.303
12	Os11g0535600	1.173	1.019	1.172	1.068	1.090	0.938	0.960	1.166	1.045	1.025	1.026	1.042
13	Os04g0649600	1.172	1.168	1.234	1.660	1.564	1.654	1.121	1.217	1.053	1.645	1.665	1.608
14	Os10g0519750	1.168	1.249	1.245	1.183	1.102	1.184	1.179	1.162	1.247	1.106	1.221	1.350
15	Os05g0494700	1.165	1.149	1.213	1.177	1.165	1.126	1.064	1.047	1.139	1.120	1.166	1.186
16	Os04g0451700	1.164	1.213	1.136	0.280	0.457	0.451	1.167	1.275	1.074	0.394	0.413	0.533
17	Os05g0209500	1.150	1.348	1.325	1.333	1.480	1.393	1.551	1.551	1.593	1.602	1.654	1.702
18	Os02g0279800	1.149	1.019	1.148	1.288	1.311	1.170	0.954	1.137	1.076	1.270	1.275	1.339
19	Os03g0288700	1.149	1.227	1.062	1.160	1.107	1.107	1.015	1.050	1.011	1.106	1.256	1.196
20	Os12g0111600	1.145	1.140	1.203	1.304	1.260	1.263	1.302	1.213	1.243	1.074	1.046	1.098
21	Os07g0690200	1.143	1.137	1.118	1.049	1.046	1.071	0.997	1.130	1.007	0.962	1.189	1.034
22	Os02g0288200	1.139	1.163	1.157	1.184	1.144	1.187	1.013	1.026	1.093	1.178	1.243	1.234
23	Os10g0501000	0.825	0.832	0.851	0.661	0.660	0.732	0.740	0.797	0.846	0.715	0.699	0.694
24	Os02g0103900	0.817	0.849	0.855	0.953	0.964	1.073	0.831	0.748	0.733	0.884	1.000	0.937
25	Os04g0407500	0.817	0.726	0.859	1.040	1.177	0.998	0.814	0.889	0.848	1.103	1.153	1.012
26	Os04g0464600	0.807	0.773	0.829	0.578	0.615	0.566	0.702	0.633	0.610	0.705	0.700	0.567
27	Os04g0668000	0.805	0.848	0.671	0.736	0.791	0.795	0.696	0.496	0.724	0.709	0.649	0.723
28	Os07g0564200	0.798	1.027	0.807	0.552	0.579	0.716	1.103	0.947	0.828	0.687	0.801	0.852
29	Os06g0543400	0.798	0.967	0.717	0.805	0.840	0.724	0.730	0.758	0.739	0.805	0.710	0.750

Table S3. *Cont.*

No.	Gene ID	Sample names											
		WT0_1	WT0_2	WT0_3	WT24_1	WT24_2	WT24_3	RI1-2_0_1	RI1-2_0_2	RI1-2_0_3	RI1-2_24_1	RI1-2_24_2	RI1-2_24_3
30	Os01g0687400	0.797	0.757	0.741	2.363	2.326	2.262	1.287	1.072	1.270	2.374	2.367	2.431
31	Os01g0266100	0.795	0.864	0.812	0.846	0.911	0.863	0.617	0.873	0.705	0.872	0.899	0.964
32	Os10g0562000	0.795	0.932	0.789	0.953	0.882	0.807	0.861	0.662	0.752	0.860	0.772	0.655
33	Os06g0119100	0.793	0.772	0.757	0.293	0.267	0.289	0.700	0.522	0.519	0.310	0.328	0.262
34	Os06g0499100	0.793	0.811	0.937	1.575	1.650	1.643	0.793	0.701	0.528	1.672	1.536	1.691
35	Os03g0682100	0.792	0.585	0.705	0.159	0.452	0.328	0.687	0.621	0.647	0.398	0.515	0.302
36	Os03g0124200	0.789	0.677	0.700	0.847	0.785	0.831	0.787	0.690	0.773	0.833	0.890	0.938
37	Os02g0108800	0.788	0.706	0.929	0.843	0.806	1.001	0.789	0.618	0.848	0.934	1.052	0.876
38	Os03g0156700	0.783	0.900	0.796	1.610	1.603	1.564	0.813	0.677	0.648	1.549	1.488	1.540
39	Os01g0793900	0.783	0.846	0.917	1.080	1.176	1.116	0.853	0.927	0.792	1.082	1.140	1.129
40	Os01g0759550	0.781	0.757	0.878	0.486	0.419	0.470	0.675	0.648	0.707	0.508	0.508	0.332
41	Os01g0181166	0.773	0.849	0.980	1.012	0.872	1.045	1.014	0.930	0.851	0.949	0.884	0.984
42	Os07g0200900	0.771	0.825	0.758	0.886	0.981	1.009	0.842	0.940	0.915	0.949	0.940	0.955
43	Os09g0529900	0.767	0.746	0.784	0.760	0.881	0.720	0.743	0.653	0.736	0.664	0.823	0.718
44	Os07g0604800	0.767	0.853	0.722	0.927	0.863	0.895	0.639	0.557	0.589	0.963	0.896	0.940
45	Os01g0512200	0.766	0.781	0.749	0.759	0.792	0.811	0.727	0.692	0.773	0.816	0.775	0.827
46	Os04g0415401	0.758	0.830	0.932	0.970	1.026	0.946	0.643	0.718	0.816	0.818	0.828	0.858
47	Os02g0258000	0.748	0.689	0.730	0.514	0.472	0.478	0.763	0.840	0.628	0.599	0.538	0.584
48	Os03g0800400	0.748	0.594	0.739	0.550	0.659	0.562	0.573	0.605	0.710	0.629	0.542	0.619
49	Os03g0569850	0.748	0.684	0.762	0.202	0.232	0.250	0.755	0.821	0.863	0.219	0.292	0.245
50	Os03g0356526	0.746	0.906	0.620	1.070	0.979	0.991	0.753	0.695	0.681	1.073	1.212	1.140
51	Os06g0610600	0.745	0.494	0.698	0.798	0.791	0.743	0.413	0.446	0.414	0.846	0.935	0.973
52	Os01g0826800	0.744	0.900	0.750	0.542	0.561	0.333	0.594	0.730	0.896	0.476	0.564	0.489
53	Os07g0585900	0.742	0.554	0.600	1.149	1.214	1.195	0.695	0.847	0.925	1.204	1.324	1.319
54	Os06g0584200	0.741	0.674	0.772	0.744	0.733	0.735	0.731	0.645	0.704	0.725	0.729	0.773
55	Os02g0567800	0.737	0.522	0.695	0.596	0.662	0.605	0.661	0.542	0.402	0.595	0.678	0.754
56	Os07g0598500	0.731	0.704	0.731	0.702	0.643	0.610	0.716	0.707	0.635	0.606	0.612	0.527
57	Os04g0685400	0.730	0.666	0.755	1.289	1.413	1.409	0.564	0.534	0.647	1.472	1.462	1.531
58	Os04g0669475	0.730	0.832	0.856	1.229	1.176	1.270	0.861	0.924	0.942	1.242	1.342	1.321

Table S3. *Cont.*

No.	Gene ID	Sample names											
		WT0_1	WT0_2	WT0_3	WT24_1	WT24_2	WT24_3	RI1-2_0_1	RI1-2_0_2	RI1-2_0_3	RI1-2_24_1	RI1-2_24_2	RI1-2_24_3
59	Os01g0523000	0.728	0.742	0.608	0.583	0.847	0.641	0.531	0.495	0.633	0.665	0.688	0.664
60	Os02g0666700	0.727	0.795	0.875	0.798	0.903	0.604	0.733	0.909	0.744	0.833	0.703	0.583
61	Os03g0149800	0.726	0.849	0.751	0.810	0.726	0.688	0.526	0.638	0.625	0.857	0.942	0.914
62	Os11g0117300	0.723	0.951	0.619	0.974	0.975	0.965	0.824	0.694	0.593	1.091	1.010	1.167
63	Os07g0423000	0.722	0.765	0.869	0.841	0.796	0.672	0.624	0.680	0.583	0.745	0.661	0.688
64	Os12g0566600	0.716	0.784	0.729	0.081	0.152	0.225	0.628	0.626	0.627	0.114	0.290	0.148
65	Os05g0403400	0.715	0.823	0.929	0.848	0.788	0.796	0.730	0.728	0.704	0.849	0.852	0.832
66	Os02g0829800	0.712	0.781	0.812	0.646	0.661	0.650	0.714	0.659	0.698	0.531	0.730	0.526
67	Os01g0795766	0.712	0.800	0.750	0.914	0.902	1.025	0.767	0.809	0.684	0.896	0.824	0.984
68	Os04g0544100	0.709	0.736	0.728	0.662	0.648	0.687	0.615	0.722	0.738	0.695	0.757	0.691
69	Os01g0604100	0.709	0.681	0.765	0.692	0.654	0.733	0.734	0.723	0.787	0.664	0.771	0.695
70	Os05g0395400	0.703	0.628	0.666	0.653	0.623	0.670	0.779	0.800	0.729	0.501	0.576	0.467
71	Os02g0563800	0.697	0.682	0.716	0.718	0.717	0.729	0.661	0.634	0.689	0.709	0.735	0.731
72	Os04g0413900	0.693	0.631	0.682	0.737	0.728	0.842	0.752	0.906	0.766	0.755	0.882	0.850
73	Os08g0203900	0.692	0.636	0.684	0.802	0.808	0.773	0.622	0.528	0.555	0.721	0.687	0.745
74	Os03g0806700	0.691	0.591	0.568	0.462	0.513	0.405	0.535	0.595	0.550	0.586	0.394	0.374
75	Os02g0297200	0.676	0.567	0.609	1.093	0.992	1.026	0.571	0.705	0.705	1.122	1.197	1.170
76	Os01g0714100	0.675	0.714	0.701	0.751	0.726	0.791	0.642	0.677	0.605	0.764	0.757	0.766
77	Os07g0244400	0.672	0.618	0.673	0.531	0.368	0.427	0.637	0.750	0.656	0.521	0.606	0.528
78	Os01g0697100	0.671	0.625	0.580	0.951	0.909	0.869	0.665	0.639	0.537	1.000	1.033	1.017
79	Os06g0352900	0.669	0.645	0.727	0.556	0.471	0.544	0.641	0.627	0.621	0.343	0.490	0.401
80	Os12g0533500	0.666	0.642	0.657	0.651	0.655	0.640	0.571	0.641	0.542	0.612	0.662	0.599
81	Os01g0280400	0.650	0.607	0.686	0.292	0.179	0.192	0.636	0.686	0.587	0.123	0.119	0.152
82	Os04g0658100	0.640	0.633	0.667	0.711	0.640	0.658	0.635	0.615	0.663	0.690	0.695	0.667
83	Os05g0391200	0.635	0.711	0.655	0.563	0.607	0.604	0.620	0.551	0.560	0.655	0.525	0.568
84	Os03g0295866	0.578	0.528	0.561	0.678	0.591	0.516	0.593	0.474	0.358	0.473	0.367	0.341
85	Os04g0477200	0.570	0.456	0.592	0.496	0.460	0.390	0.530	0.516	0.516	0.406	0.434	0.408
86	Os04g0433900	0.029	0.009	0.014	0.014	0.003	0.012	0.007	0.031	0.011	0.024	0.013	0.006
87	Os11g0515700	0.027	0.050	0.014	0.212	0.216	0.171	0.027	0.050	0.085	0.169	0.139	0.215

Table S3. *Cont.*

No.	Gene ID	Sample names											
		WT0_1	WT0_2	WT0_3	WT24_1	WT24_2	WT24_3	RI1-2_0_1	RI1-2_0_2	RI1-2_0_3	RI1-2_24_1	RI1-2_24_2	RI1-2_24_3
88	Os09g0512000	0.027	0.009	0.026	0.021	0.021	0.018	0.009	0.027	0.039	0.015	0.016	0.032
89	Os11g0579900	0.024	0.042	0.035	0.028	0.015	0.043	0.055	0.051	0.017	0.036	0.015	0.015
90	Os01g0553901	0.023	0.032	0.020	0.064	0.069	0.046	0.052	0.023	0.085	0.103	0.090	0.158
91	Os01g0369200	0.022	0.017	0.042	0.016	0.053	0.014	0.069	0.031	0.034	0.022	0.012	0.022
92	Os11g0448100	0.017	0.025	0.011	0.007	0.031	0.025	0.017	0.011	0.012	0.010	0.018	0.014
93	Os11g0481600	0.016	0.038	0.024	0.000	0.010	0.016	0.008	0.000	0.009	0.007	0.015	0.010
94	Os03g0258500	0.015	0.049	0.029	0.009	0.036	0.023	0.052	0.036	0.000	0.037	0.027	0.045
95	Os04g0210250	0.014	0.039	0.040	0.016	0.006	0.005	0.005	0.009	0.015	0.023	0.029	0.011
96	Os11g0641500	0.014	0.039	0.014	0.796	0.663	0.567	0.029	0.131	0.234	0.944	0.805	0.826
97	Os07g0152301	0.012	0.010	0.008	0.002	0.010	0.006	0.035	0.048	0.042	0.005	0.009	0.019
98	Os04g0138432	0.007	0.048	0.022	0.073	0.091	0.093	0.202	0.342	0.282	0.090	0.088	0.112
99	Os02g0179250	0.007	0.014	0.005	0.003	0.006	0.005	0.002	0.002	0.005	0.002	0.004	0.003
100	Os11g0476333	0.003	0.022	0.011	0.026	0.030	0.020	0.006	0.009	0.012	0.038	0.027	0.033

Table S4. Gene ontology (GO) enrichment analysis of up-regulated differentially expressed genes (DEGs) in *OsVTC1-1* RI1-2 line between 0 and 24 h after rice blast inoculation. The GO categories include biological process (BP), cellular component (CC), and molecular function (MF).

No.	GO term	GO ID	Category	Count	*p*-value
1	cellular process	GO:0009987	BP	331	7.47E-07
2	metabolic process	GO:0008152	BP	259	2.10E-04
3	biological regulation	GO:0065007	BP	142	2.13E-05
4	response to stimulus	GO:0050896	BP	134	2.21E-10
5	regulation of biological process	GO:0050789	BP	134	1.30E-06
6	regulation of cellular process	GO:0050794	BP	126	2.95E-07
7	phosphorus metabolic process	GO:0006793	BP	86	2.54E-05
8	phosphate-containing compound metabolic process	GO:0006796	BP	81	1.89E-04
9	cellular response to stimulus	GO:0051716	BP	66	5.16E-05
10	cell communication	GO:0007154	BP	52	5.31E-06
11	response to chemical	GO:0042221	BP	47	3.07E-05
12	signaling	GO:0023052	BP	46	1.49E-05
13	signal transduction	GO:0007165	BP	46	8.70E-06
14	response to organic substance	GO:0010033	BP	34	4.71E-05
15	regulation of response to stimulus	GO:0048583	BP	19	3.61E-05
16	nucleic acid metabolic process	GO:0090304	BP	17	3.98E-05
17	regulation of response to stress	GO:0080134	BP	14	9.82E-06
18	regulation of defense response	GO:0031347	BP	10	5.03E-05
19	nucleotide-sugar metabolic process	GO:0009225	BP	7	9.71E-05
20	cellular anatomical entity	GO:0110165	CC	460	4.00E-11
21	intracellular anatomical structure	GO:0005622	CC	287	3.04E-07
22	intracellular organelle	GO:0043229	CC	242	1.28E-05
23	organelle	GO:0043226	CC	242	1.54E-05
24	membrane	GO:0016020	CC	238	1.06E-05
25	intracellular membrane-bounded organelle	GO:0043231	CC	227	2.02E-05
26	membrane-bounded organelle	GO:0043227	CC	227	2.47E-05
27	intrinsic component of membrane	GO:0031224	CC	196	4.98E-04
28	cytoplasm	GO:0005737	CC	193	1.84E-06
29	integral component of membrane	GO:0016021	CC	192	6.42E-04
30	cell periphery	GO:0071944	CC	61	4.01E-04
31	plasma membrane	GO:0005886	CC	56	2.71E-04
32	endomembrane system	GO:0012505	CC	54	9.72E-04
33	cytosol	GO:0005829	CC	43	1.52E-05
34	bounding membrane of organelle	GO:0098588	CC	33	2.11E-05
35	nuclear protein-containing complex	GO:0140513	CC	2	5.98E-05
36	nuclear lumen	GO:0031981	CC	1	2.35E-04
37	binding	GO:0005488	MF	311	4.00E-11
38	catalytic activity	GO:0003824	MF	284	6.82E-09
39	organic cyclic compound binding	GO:0097159	MF	213	2.31E-08
40	heterocyclic compound binding	GO:1901363	MF	212	1.31E-05
41	ion binding	GO:0043167	MF	183	1.93E-05
42	transferase activity	GO:0016740	MF	130	6.92E-05
43	DNA binding	GO:0003677	MF	71	1.83E-05
44	DNA-binding transcription factor activity	GO:0003700	MF	46	1.96E-04
45	sequence-specific DNA binding	GO:0043565	MF	41	1.84E-05

Table S5. Plant reactome pathway analysis of up-regulated differentially expressed genes (DEGs) in *OsVTC1-1* RI1-2 line between 0 and 24 h after rice blast inoculation.

No.	Pathway	Reactome ID	Count
1	Metabolism and regulation	R-OSA-2744345	62
2	Hormone signaling, transport, and metabolism	R-OSA-2744341	29
3	Carbohydrate metabolism	R-OSA-2883407	14
4	Growth and developmental processes	R-OSA-9030769	13
5	Reproductive structure development	R-OSA-9031669	12
6	Seed development	R-OSA-9623902	11
7	Regulation of seed size	R-OSA-9035605	10
8	Amino acid metabolism	R-OSA-2744343	8
9	Jasmonic acid signaling	R-OSA-6787011	8
10	Salicylic acid signaling	R-OSA-6788019	7
11	Fatty acid and lipid metabolism	R-OSA-3906998	6
12	Secondary metabolism	R-OSA-2744344	6
13	Ethylene biosynthesis and signaling	R-OSA-5225808	5
14	Auxin signaling	R-OSA-5608118	5
15	Ethylene biosynthesis from methionine	R-OSA-1119334	4
16	UDP-L-arabinose biosynthesis and transport	R-OSA-1119574	4
17	Responses to stimuli: abiotic stimuli and stresses	R-OSA-8932729	4
18	Methionine salvage pathway	R-OSA-1119624	3
19	Arsenic uptake and detoxification	R-OSA-9618218	3
20	Phospholipid biosynthesis I	R-OSA-1119402	3
21	Response to heavy metals	R-OSA-9618278	3
22	Calvin cycle	R-OSA-1119519	3
23	Inorganic nutrients metabolism	R-OSA-5368291	3
24	G1/S transition	R-OSA-9640887	3
25	Brassinosteroid signaling	R-OSA-5632095	3
26	Mitosis	R-OSA-9640713	3
27	Cell cycle	R-OSA-9640670	3
28	Cellular processes	R-OSA-2894886	3
29	Amino acid biosynthesis	R-OSA-5655122	3
30	S-adenosyl-L-methionine cycle	R-OSA-1119501	2
31	UDP-D-xylose biosynthesis	R-OSA-1119563	2
32	Mugineic acid biosynthesis	R-OSA-9025754	2
33	Ureide biosynthesis	R-OSA-1119407	2
34	GDP-D-rhamnose biosynthesis	R-OSA-1119428	2
35	GDP-L-fucose biosynthesis I (from GDP-D-mannose)	R-OSA-1119620	2
36	Nucleotide metabolism	R-OSA-5655149	2
37	G1 phase	R-OSA-9640760	2
38	Galactose degradation II	R-OSA-1119452	2
39	Response to iron deficiency	R-OSA-9025714	2
40	Sucrose biosynthesis	R-OSA-1119465	2
41	TCA cycle (plant)	R-OSA-1119533	2
42	Generation of precursor metabolites and energy	R-OSA-2961031	2
43	Cytosolic glycolysis	R-OSA-1119570	2
44	Amino acid catabolism	R-OSA-5655124	2
45	Phytocassane biosynthesis	R-OSA-1119583	1
46	Coumarin biosynthesis (via 2-coumarate)	R-OSA-1119284	1
47	Response to phosphate deficiency	R-OSA-9031225	1
48	Mannose degradation	R-OSA-1119595	1
49	Metabolism and regulation	R-OSA-1119628	1

Table S5. *Cont.*

No.	Pathway	Reactome ID	Count
50	Cardiolipin biosynthesis	R-OSA-1119260	1
51	Lysine degradation II	R-OSA-1119365	1
52	Glutamate degradation	R-OSA-1119458	1
53	13-LOX and 13-HPL pathway	R-OSA-1119618	1
54	Divinyl ether biosynthesis II (13-LOX)	R-OSA-1119566	1
55	Linear furanocoumarin biosynthesis	R-OSA-1119353	1
56	Glycolipid desaturation	R-OSA-1119300	1
57	Cellulose biosynthesis	R-OSA-1119314	1
58	Phenylpropanoid biosynthesis, initial reactions	R-OSA-1119582	1
59	Cyanate degradation	R-OSA-1119586	1
60	Ethylene mediated signaling	R-OSA-5225756	1
61	Suberin biosynthesis	R-OSA-1119418	1
62	Mevalonate pathway	R-OSA-1119615	1
63	Primary root development	R-OSA-9030654	1
64	Leucine biosynthesis	R-OSA-1119540	1
65	Tryptophan biosynthesis	R-OSA-1119494	1
66	Chorismate biosynthesis	R-OSA-1119430	1
67	Choline biosynthesis III	R-OSA-1119276	1
68	Jasmonic acid biosynthesis	R-OSA-1119332	1
69	Ascorbate biosynthesis	R-OSA-1119410	1
70	Flower development	R-OSA-9609102	1
71	Starch biosynthesis	R-OSA-1119477	1
72	Sphingolipid metabolism	R-OSA-1119325	1
73	Abscisic acid (ABA) mediated signaling	R-OSA-3899351	1
74	HSFA7/ HSFA6B-regulatory network-induced by drought and ABA	R-OSA-9623703	1
75	Response to salinity	R-OSA-9638808	1
76	Response to drought	R-OSA-9623744	1
77	Cytokinins-O-glucoside biosynthesis	R-OSA-1119473	1
78	ABA biosynthesis and mediated signaling	R-OSA-3899368	1
79	Regulatory network of nutrient accumulation	R-OSA-9626305	1
80	Root structure development	R-OSA-9640032	1
81	Vegetative structure development	R-OSA-9031670	1
82	Cofactor biosyntheses	R-OSA-2867929	1

Table S6. The top 10 gene ontology (GO) enrichment analysis of differentially expressed proteins (DEPs) in *OsVTC1-1* RI1-2 line at 24 h after inoculation. The GO categories include biological process (BP), cellular component (CC), and molecular function (MF).

No.	GO term	GO ID	Category	Count	*p*-value
1	cellular process	GO:0009987	BP	110	4.04E-32
2	metabolic process	GO:0008152	BP	82	4.04E-18
3	organic substance metabolic process	GO:0071704	BP	78	6.60E-18
4	cellular metabolic process	GO:0044237	BP	71	1.25E-14
5	primary metabolic process	GO:0044238	BP	67	1.24E-12
6	nitrogen compound metabolic process	GO:0006807	BP	54	3.32E-09
7	macromolecule metabolic process	GO:0043170	BP	39	4.89E-04
8	organonitrogen compound metabolic process	GO:1901564	BP	37	2.17E-05
9	biological regulation	GO:0065007	BP	35	5.95E-06
10	response to stimulus	GO:0050896	BP	34	2.58E-08
11	cellular anatomical entity	GO:0110165	CC	116	1.91E-23
12	intracellular anatomical structure	GO:0005622	CC	97	6.72E-28
13	organelle	GO:0043226	CC	85	1.29E-23
14	intracellular organelle	GO:0043229	CC	85	1.23E-23
15	membrane-bounded organelle	GO:0043227	CC	79	3.48E-21
16	intracellular membrane-bounded organelle	GO:0043231	CC	79	3.00E-21
17	cytoplasm	GO:0005737	CC	72	2.39E-22
18	nucleus	GO:0005634	CC	33	1.02E-05
19	plastid	GO:0009536	CC	23	6.76E-12
20	chloroplast	GO:0009507	CC	23	2.18E-12
21	binding	GO:0005488	MF	97	9.06E-26
22	catalytic activity	GO:0003824	MF	89	5.54E-23
23	ion binding	GO:0043167	MF	70	5.70E-21
24	organic cyclic compound binding	GO:0097159	MF	66	8.86E-15
25	heterocyclic compound binding	GO:1901363	MF	66	8.32E-15
26	small molecule binding	GO:0036094	MF	45	1.08E-14
27	anion binding	GO:0043168	MF	41	2.08E-12
28	nucleoside phosphate binding	GO:1901265	MF	41	8.25E-13
29	nucleotide binding	GO:0000166	MF	41	8.25E-13
30	cation binding	GO:0043169	MF	40	9.66E-13

Table S7. Plant reactome pathway analysis of differentially expressed proteins (DEPs) in *OsVTC1-1* RI1-2 line at 24 h after inoculation.

No.	Pathway	Reactome ID	Count
1	Metabolism and regulation	R-OSA-2744345	28
2	Hormone signaling, transport, and metabolism	R-OSA-2744341	12
3	Growth and developmental processes	R-OSA-9030769	6
4	Reproductive structure development	R-OSA-9031669	5
5	Amino acid metabolism	R-OSA-2744343	4
6	Secondary metabolism	R-OSA-2744344	4
7	Seed development	R-OSA-9623902	4
8	Regulation of seed size	R-OSA-9035605	4
9	Cofactor biosyntheses	R-OSA-2867929	3
10	Auxin signaling	R-OSA-5608118	3
11	Amino acid biosynthesis	R-OSA-5655122	3
12	Carbohydrate metabolism	R-OSA-2883407	3
13	GA12 biosynthesis	R-OSA-1119557	2
14	Biotin biosynthesis II	R-OSA-1119610	2
15	Fatty acid and lipid metabolism	R-OSA-3906998	2
16	Oleoresin sesquiterpene volatiles biosynthesis	R-OSA-1119328	1
17	Ent-kaurene biosynthesis	R-OSA-1119348	1
18	Momilactone biosynthesis	R-OSA-1119308	1
19	Canavanine biosynthesis	R-OSA-1119444	1
20	IAA biosynthesis II	R-OSA-1119580	1
21	Glutamate degradation	R-OSA-1119458	1
22	UDP-N-acetylgalactosamine biosynthesis	R-OSA-1119386	1
23	Gibberellin biosynthesis III (early C-13 hydroxylation)	R-OSA-1119294	1
24	Anther and pollen development	R-OSA-8986768	1
25	Removal of superoxide radicals	R-OSA-1119403	1
26	Pantothenate biosynthesis I	R-OSA-1119496	1
27	Pantothenate biosynthesis II	R-OSA-1119544	1
28	Plastid glycolysis	R-OSA-1119424	1
29	GDP-D-rhamnose biosynthesis	R-OSA-1119428	1
30	GDP-L-fucose biosynthesis I (from GDP-D-mannose)	R-OSA-1119620	1
31	Polar auxin transport	R-OSA-8858053	1
32	Primary root development	R-OSA-9030654	1
33	Leucine biosynthesis	R-OSA-1119540	1
34	Galactose degradation II	R-OSA-1119452	1
35	Chorismate biosynthesis	R-OSA-1119430	1
36	Reactive oxygen species (ROS) homeostasis	R-OSA-9607141	1
37	Detoxification	R-OSA-9613003	1
38	Gibberellin signaling	R-OSA-5679411	1
39	Phospholipid biosynthesis I	R-OSA-1119402	1
40	UDP-L-arabinose biosynthesis and transport	R-OSA-1119574	1
41	Sucrose biosynthesis	R-OSA-1119465	1
42	Auxin transport	R-OSA-8868860	1
43	Sphingolipid metabolism	R-OSA-1119325	1
44	TCA cycle (plant)	R-OSA-1119533	1
45	Phenylpropanoid biosynthesis	R-OSA-1119316	1
46	Generation of precursor metabolites and energy	R-OSA-2961031	1
47	Root structure development	R-OSA-9640032	1
48	Salicylic acid signaling	R-OSA-6788019	1
49	Brassinosteroid signaling	R-OSA-5632095	1

Table S7. *Cont.*

No.	Pathway	Reactome ID	Count
50	Amino acid catabolism	R-OSA-5655124	1
51	Formation of the ternary complex, and subsequently, the 43S complex	R-OSA-1112990	1
52	Vegetative structure development	R-OSA-9031670	1
53	Jasmonic acid signaling	R-OSA-6787011	1
54	Ribosomal scanning and start codon recognition	R-OSA-1112968	1
55	Formation of a pool of free 40S subunits	R-OSA-1112971	1
56	Cap-dependent translation initiation	R-OSA-1112967	1
57	Protein metabolism: translation	R-OSA-2972871	1
58	Cellular processes	R-OSA-2894886	1
